# Clinical implications of pre-existing adenoma in endoscopically resected early gastric cancers

**DOI:** 10.1371/journal.pone.0178419

**Published:** 2017-05-25

**Authors:** Ji Min Choi, Sang Gyun Kim, Jung Kim, Seung Jun Han, Jae Yong Park, Sooyeon Oh, Jong Pil Im, Joo Sung Kim, Woo Ho Kim, Hyun Chae Jung

**Affiliations:** 1 Department of Internal Medicine, Healthcare Research Institute, Seoul National University Hospital Healthcare System Gangnam Center, Seoul, Korea; 2 Department of Internal Medicine and Liver Research Institute, Seoul National University College of Medicine, Seoul, Korea; 3 Department of Pathology, Seoul National University College of Medicine, Seoul, Korea; University Hospital Llandough, UNITED KINGDOM

## Abstract

**Background:**

Although gastric adenoma is widely accepted as a precursor of gastric cancer, pre-existing adenoma is not always detected in gastric cancer patients.

**Objective:**

To investigate the clinical characteristics of early gastric cancer (EGC) arising from adenoma, compared with those of EGC without pre-existing adenoma.

**Methods:**

Patients who underwent endoscopic resection for EGC at a single tertiary hospital were divided into two groups based on the presence (ex-adenoma group) or absence (*de novo* group) of pre-existing adenoma on pathologic specimens. Clinicopathologic characteristics, endoscopic features and long-term outcomes were analyzed.

**Results:**

Of 1,509 patients, 236 (15.6%) were included in the ex-adenoma group. Mean age (*P* = 0.003) and *Helicobacter pylori* infection rate (*P* = 0.040) were significantly higher in the ex-adenoma than in the *de novo* group. Mean endoscopic size was significantly larger, elevated lesions were more prevalent (both *P* < 0.001), and carcinomas were more differentiated in the ex-adenoma group than in the *de novo* group (*P* = 0.037). The degree of atrophy (*P* = 0.025) or intestinal metaplasia (*P* < 0.001) was more advanced in the ex-adenoma group. Synchronous gastric neoplasia was significantly more prevalent in the ex-adenoma group (P < 0.001), whereas metachronous cancer recurrence rate was not significantly different between the two groups.

**Conclusions:**

EGCs with pre-existing adenoma show a greater association with *H*. *pylori*–related chronic inflammation than those without, which could explain the differences in the characteristics between groups. Potential differences in carcinogenic mechanisms between the groups were explored.

## Introduction

Gastric cancer is the fourth most common cancer and the second leading cause of cancer-related death in the world [1, 2]. Although the incidence and mortality of gastric cancer have been decreasing worldwide, it remains one of the most common cancers in East Asia, especially in Korea and Japan [3]. Improvements in endoscopic techniques and instruments have made endoscopic submucosal dissection (ESD) the curative treatment modality of choice for early gastric cancer (EGC) without lymph node metastasis. Although ESD has shown many advantages over conventional surgery, including reduced invasiveness, lower cost, and shorter hospital stay, metachronous cancer may subsequently occur in the residual gastric mucosa [4]. Thus, regular examination for metachronous gastric cancer is necessary in patients who undergo ESD.

Although the precise mechanisms underlying gastric tumorigenesis remain unclear, it is thought to be a multifactorial and multistep process. Chronic *Helicobacter pylori* infection, together with genetic or environmental factors are major determinants of the risk of gastric cancer [5]. Since Correa *et al*. proposed the hypothesis of gastric carcinogenesis, it has been widely accepted that chronic inflammation develops into atrophic gastritis, intestinal metaplasia, gastric adenoma, and eventually gastric adenocarcinoma [5]. *H*. *pylori* infection plays an important role in the initiation of this sequential process. Gastric adenoma is considered as a significant precancerous lesion, and the annual incidence of gastric cancer is 0.6% for patients with mild to moderate dysplasia and 6% for those with severe dysplasia [6].

Carcinogenesis from adenoma to carcinoma, which is known as the adenoma-carcinoma sequence, is a well-established theory in colorectal cancer [7]. The adenoma-carcinoma sequence has also been recognized in the field of gastric cancer, although it is found less frequently than in colorectal cancer [8, 9]. Differentiated or intestinal-type carcinoma, rather than diffuse-type carcinoma, is mainly associated with this carcinogenic pathway [9, 10]. Gastric cancer derived from the adenoma-carcinoma sequence might arise through a stepwise accumulation of genetic alterations similar to that of colorectal cancer; however, other clinical characteristics of gastric cancer developed from adenoma have not been well described to date [11].

The aim of the present study was to examine the clinical characteristics of EGC arising from adenoma compared with that without pre-existing adenoma. Baseline demographics, endoscopic and pathologic features, and long-term outcomes were analyzed in detail.

## Methods

### Patients and study design

**Fig 1:**
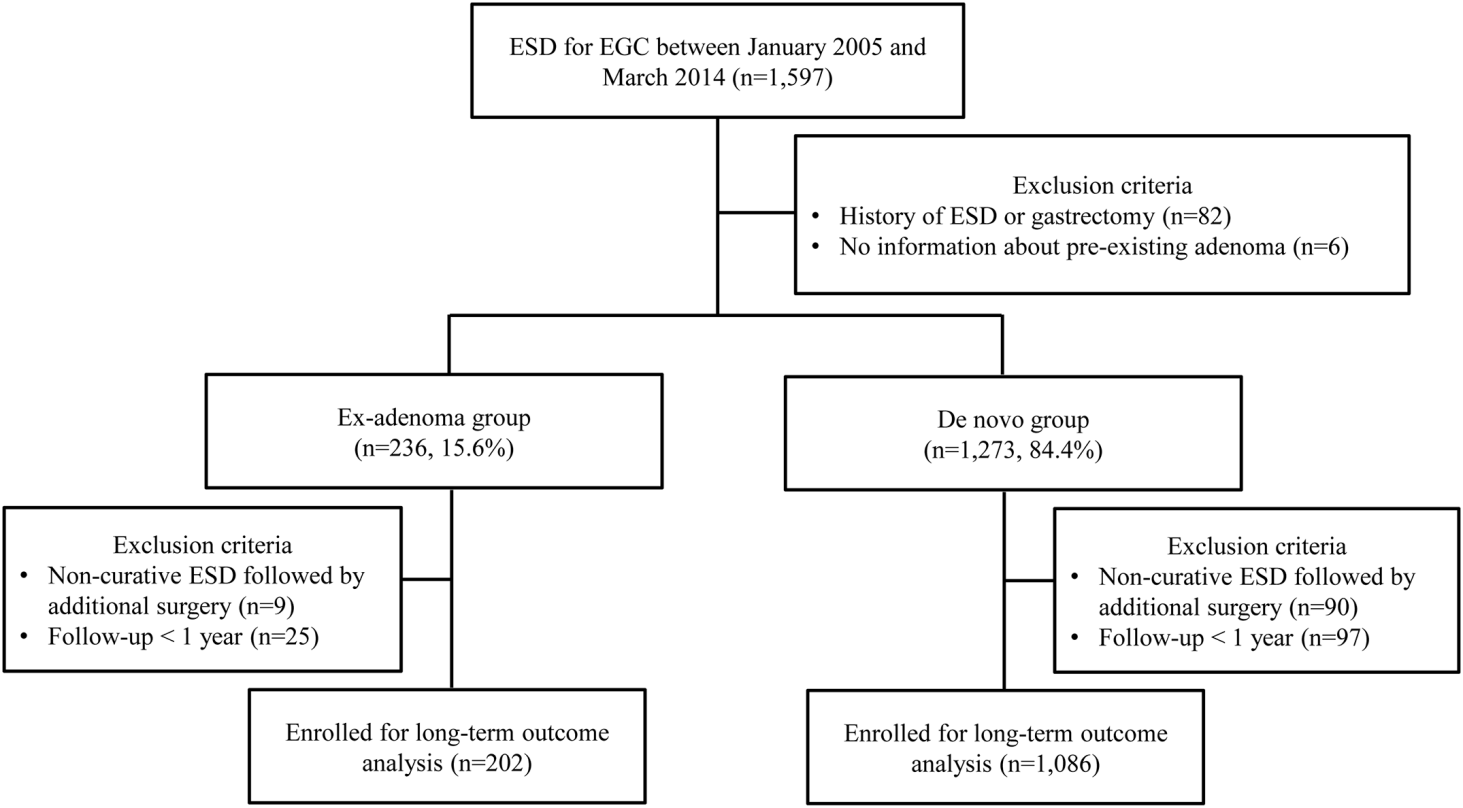
Schematic protocol of this study.

**Fig 2:**
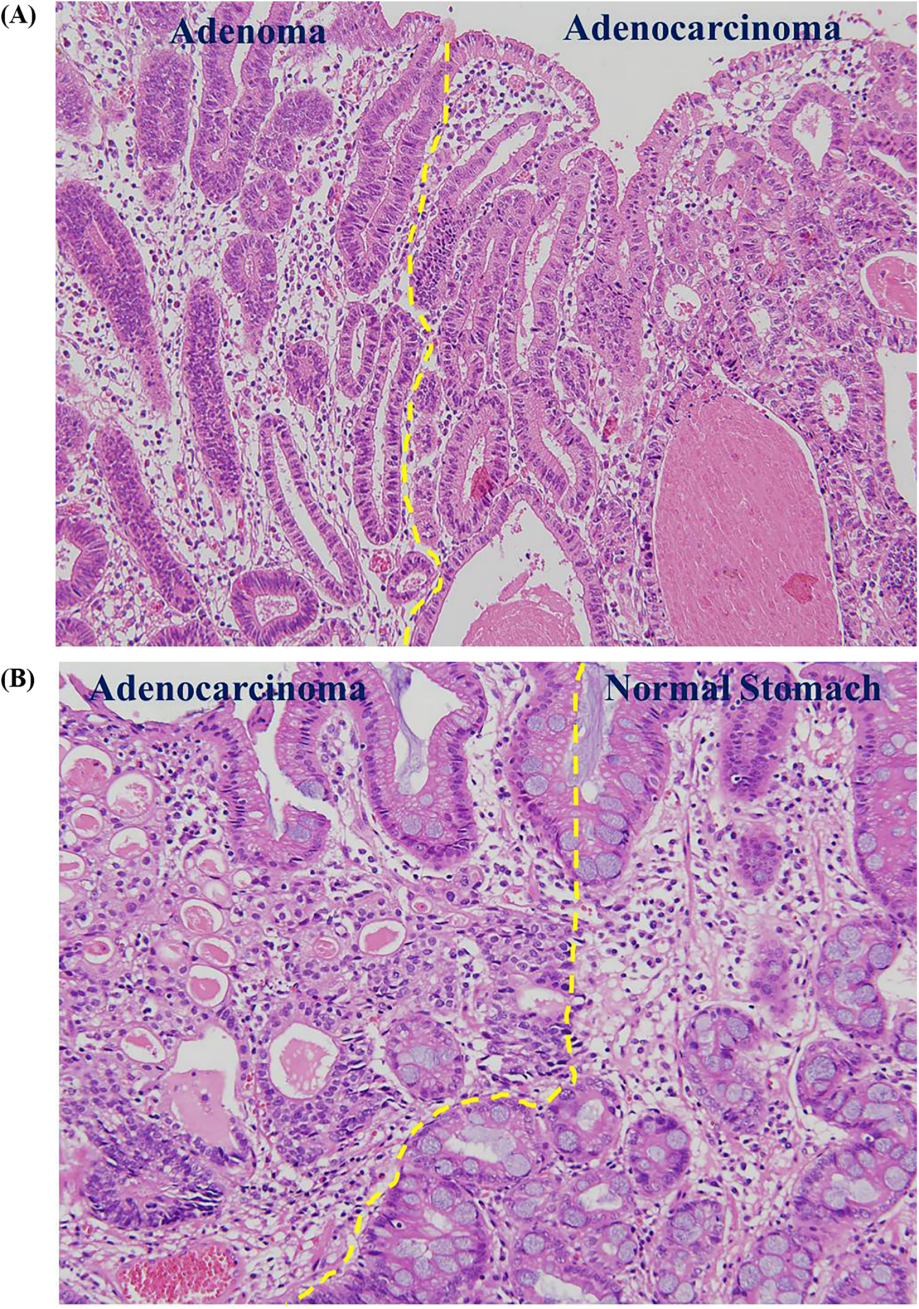
Histopathology of early gastric cancers with or without pre-existing adenoma. (A) Representative image of the ex-adenoma group. Adenomatous components (left) were detected at the margin of the tubular adenocarcinoma (right) (Hematoxylin and eosin stain; original magnification, 200×). (B) Representative image of the *de novo* group. A sharp transition from the normal gastric mucosa (right) to tubular adenocarcinoma (left) was observed. No evidence of gastric adenoma was found in its vicinity.

A schematic protocol of the study design is provided in Fig 1. Between January 2005 and March 2014, patients who underwent ESD for EGCs at Seoul National University Hospital (Seoul, Republic of Korea) were screened for this retrospective cohort study. Patients with a prior history of ESD or gastrectomy or those who had insufficient information about the presence of pre-existing adenoma were excluded from the analysis. The remaining patients were assigned to one of two groups according to the presence or absence of pre-existing adenoma. Patients with adenomatous components at the margin of EGCs were defined as the ex-adenoma group, and those without were defined as the *de novo* group (Fig 2). The medical records of the patients were reviewed with regard to age, gender, comorbidities, *H*. *pylori* positivity, and endoscopic features including size, location, macroscopic type, and gross morphology of tumors. Pathologic characteristics such as lesion size on the resected specimen, depth of invasion, cancer differentiation, Lauren’s classification, severity of atrophy, and intestinal metaplasia in the adjacent mucosa were also examined. Long-term outcomes including metachronous cancer recurrence were recorded. The study was approved by the Ethics Committee of the Seoul National University Hospital (IRB no. H-1404-106-572) and was conducted in accordance with the Declaration of Helsinki.

### ESD procedures

The indications for ESD in the treatment of EGC were defined as follows: (1) differentiated adenocarcinoma, (2) lesions ≤2 cm in diameter upon endoscopic estimation, and (3) no evidence of submucosal invasion or lymph node/distant metastasis on endoscopic ultrasonography and/or abdominal computed tomography. The ESD procedure was performed as described elsewhere [12, 13]. Intravenous midazolam (0.06 mg/kg) was administered for conscious sedation with cardiorespiratory monitoring. ESD was performed using a standard single-channel endoscope (Olympus H260; Olympus Optical Co, Tokyo, Japan) with an insulation-tipped knife (Helmet Snare; Kachu Technology Co., Seoul, Republic of Korea). After completion of ESD, biopsy samples of noncancerous gastric mucosae were obtained from two sites in the lesser curvature side of the antrum and two sites in the lesser curvature side of the body after obtaining informed consent [14].

### Evaluation of endoscopic characteristics

Endoscopic still images and reports were retrospectively reviewed to investigate the size, location, macroscopic type, and gross morphology of the lesion. Indigo carmine chromoendoscopy was used to estimate lesion size more precisely during ESD. The location of the lesion was divided into three parts: upper, middle, and lower [15]. According to the Paris endoscopic classification, lesions were classified into three macroscopic types: elevated, flat, or depressed [16]. Gross morphological features such as erythema, ulcer, erosion, fold convergence, exudate, whitish discoloration, spontaneous bleeding, or nodularity were also evaluated. An ulcer was defined as a loss of mucosal integrity (>5 mm in diameter) with a well-defined crater, whereas an erosion was defined as a flat or slightly depressed mucosal break <5 mm in diameter [17, 18]. The presence of fold convergence was characterized by abrupt cutting, clubbing, and fusion of adjacent folds. Nodularity was defined as the presence of an irregularly raised or nodular mucosa without a dominant mass [19].

### Evaluation of pathologic characteristics

Pathological diagnosis was made on the basis of the third edition of the Japanese Classification of Gastric Carcinoma: size, histologic type, and depth of invasion were evaluated [15]. In addition, histologic subtyping of gastric carcinoma was performed according to Lauren’s classification into intestinal, diffuse, and mixed types [20]. Noncancerous gastric tissues were analyzed for the severity of mucosal atrophy and intestinal metaplasia and histologically evaluated for the presence of *H*. *pylori* [21]. The severity of mucosal atrophy and intestinal metaplasia were classified as negative (absent to mild) or positive (moderate to severe). *H*. *pylori* infection status was considered positive when either rapid urease test (CLO test; Delta West, Bently, Australia) or histology was positive.

### Follow-up strategies and long-term outcome measurements

Endoscopic examinations were performed at 3, 6, and 12 months after ESD and annually thereafter to detect any residual lesion or metachronous cancer of the stomach. Metachronous gastric cancer was defined as a new carcinoma developed at a previously uninvolved site in the remnant stomach at least 1 year after ESD. Gastric neoplasms detected within 1 year of ESD were regarded as missed lesions at the initial evaluation and categorized separately as synchronous gastric neoplasias. For long-term outcome analysis, patients who underwent additional surgery immediately after ESD or those whose follow-up period was less than 1 year were excluded.

### Statistical analysis

Data were analyzed using SPSS version 21.0 (SPSS Inc., Chicago, IL, USA). All data were expressed as the mean ± standard deviation (range) or as numbers (percentages). The means of continuous variables were compared using the Student t-test, and categorical variables were analyzed with the chi-square test or Fisher’s exact test. Metachronous cancer recurrence-free survival was calculated using the Kaplan-Meier method. All unknown values were excluded from the analysis. A P-value <0.05 was considered statistically significant.

## Results

### Baseline characteristics of patients

Between January 2005 and March 2014, 1,597 patients with EGC were treated by ESD. Of these, 1,509 patients were included in the study and allocated to one of two groups based on the presence of adenomatous components at the edge of EGCs, namely the ex-adenoma group (n = 236) and the *de novo* group (n = 1,273). The baseline characteristics of the two groups are shown in Table 1. The mean age was significantly higher in the ex-adenoma group than in the *de novo* group (65.61 ± 9.22 vs. 63.62 ± 9.62 years, *P* = 0.003). The proportion of male patients, body mass index, and medical comorbidities were not significantly different between the two groups. *H*. *pylori* infection rate was higher in the ex-adenoma group than in the *de novo* group (64.8% vs. 57.2%, *P* = 0.040).

**Table 1:** Baseline characteristics of patients with early gastric cancers treated with endoscopic submucosal dissection.

	Ex-adenoma group (n = 236)	De novo group (n = 1273)	*P* value
**Mean age, year**	65.61 ± 9.22	63.62 ± 9.62	0.003
**Gender, male**	157 (66.5)	875 (68.7)	0.502
**Body mass index, mean**	24.04 ± 3.23	24.44 ± 8.96	0.500
**Comorbidities**			
Diabetes mellitus	34 (14.4)	160 (12.6)	0.438
Hypertension	69 (29.2)	353 (27.7)	0.636
Lung disease	9 (3.8)	28 (2.2)	0.141
Chronic liver disease	15 (6.4)	66 (5.2)	0.463
Chronic kidney disease	3 (1.3)	15 (1.2)	0.904
Stroke	3 (1.3)	38 (3.0)	0.137
Coronary heart disease	8 (3.4)	70 (5.5)	0.179
Other malignancy	17 (7.2)	87 (6.8)	0.837
***H*. *pylori* infection**^a^			0.040
Positive	138 (64.8)	680 (57.2)	
Negative	75 (35.2)	508 (42.8)	
Unknown^b^	23 (—)	85 (—)	

Values are presented as mean ± standard deviation or n (%)

*H*. *pylori*, *Helicobacter pylori*

^a^Patients were classified as *H*. *pylori* positive when either rapid urease test or histology was positive

^**b**^Excluded from the analysis

Additional analysis of *H*. *pylori* infection status was performed to minimize false-negative results (Table 2). As the infection progressed, the gastric mucosa showed severe atrophic changes and the bacterial load could be reduced in the stomach [22]. Patients were therefore divided into four groups based on the combination of the results of *H*. *pylori* infection and the severity of mucosal atrophy as follows: group A [*H*. *pylori* (-) and negative atrophy], group B [*H*. *pylori* (+) and negative atrophy], group C [*H*. *pylori* (+) and positive atrophy], and group D [*H*. *pylori* (-) and positive atrophy]. In this second analysis, group A patients were negative for *H*. *pylori* infection, whereas B, C, and D were considered *H*. *pylori* confirmed or expected. Group D was included as *H*. *pylori* confirmed or expected because atrophy detected in this group was considered as *H*. *pylori*–related. The *H*. *pylori* infection rate was significantly higher in the ex-adenoma group (83.4% vs. 74.2%, *P* = 0.005).

**Table 2:** Further analysis of *H*. *pylori* infection considering the severity of atrophic gastritis.

	Ex-adenoma group (n = 236)	De novo group (n = 1,273)	*P* value
***H*. *pylori* infection**^a^			0.005
*H*. *pylori* negative (Group A)	34 (16.6)	298 (25.8)	
*H*. *pylori* confirmed or expected (Group B, C, D)	171 (83.4)	856 (74.2)	
Unknown^b^	31 (—)	119 (—)	

Values are presented as n (%)

*H*. *pylori*, *Helicobacter pylori*

^a^Patients were divided into 4 groups according to the results of *H*. *pylori* infection (rapid urease test or histology) and the severity of mucosal atrophy: group A [*H*. *pylori* (-) and negative atrophy], group B [*H*. *pylori* (+) and negative atrophy], group C [*H*. *pylori* (+) and positive atrophy], and group D [*H*. *pylori* (-) and positive atrophy].

^**b**^Excluded from the analysis

### Endoscopic features of the two groups

Table 3 shows the endoscopic features of EGCs in the two groups. The lesion size estimated by endoscopy was significantly greater (19.25 ± 14.42 vs. 12.06 ± 7.49, *P* < 0.001) and the elevated type was more common in the ex-adenoma group (60.2% vs. 29.8%, *P* < 0.001) than in the *de novo* group. Erythema and erosion were significantly more frequent in the *de novo* group, whereas exudate and nodularity were more frequently observed in the ex-adenoma group. The location of the lesions did not differ between the groups.

**Table 3:** Comparison of endoscopic characteristics of early gastric cancers between the two groups.

	Ex-adenoma group (n = 236)	De novo group (n = 1,273)	*P* value
**Endoscopic size, mm**	19.25 ± 14.42	12.06 ± 7.49	< 0.001
**Location**			0.130
Upper	10 (4.2)	62 (4.9)	
Middle	88 (37.3)	390 (30.6)	
Lower	138 (58.5)	821 (64.5)	
**Macroscopic type**			< 0.001
Elevated	142 (60.2)	379 (29.8)	
Flat	30 (12.7)	262 (20.6)	
Depressed	64 (27.1)	632 (49.5)	
**Gross morphology**			
Erythema	41 (17.4)	464 (36.4)	< 0.001
Ulcer	3 (1.9)	30 (3.3)	0.050
Erosion	53 (22.5)	463 (36.4)	< 0.001
Fold convergence	2 (0.8)	14 (1.1)	1.000
Exudate	14 (5.9)	34 (2.7)	0.009
Whitish discoloration	4 (1.7)	15 (1.2)	0.522
Spontaneous bleeding	15 (6.4)	98 (7.7)	0.472
Nodularity	67 (28.4)	153 (12.0)	< 0.001

Values are presented as mean ± standard deviation or n (%)

### Pathologic characteristics of the two groups

Table 4 shows the pathologic findings of the two groups. Similar to the endoscopic size, pathologic size was significantly greater in the ex-adenoma group (23.99 ± 15.57 vs. 17.50 ± 10.88, *P* < 0.001). The presence of atrophy (47.7% vs. 39.3%, *P* = 0.025) and intestinal metaplasia (84.0% vs. 65.4%, *P* < 0.001) were significantly more frequent in the ex-adenoma group. In addition, carcinomas showed better differentiation in the ex-adenoma group (97.9% vs. 94.7%, *P* = 0.037). The depth of the lesions and Lauren’s classification did not differ significantly between the two groups.

**Table 4:** Comparison of pathologic features between the two groups.

	Ex-adenoma group (n = 236)	De novo group (n = 1,273)	*P* value
**Pathologic size, mm**	23.99 ± 15.57	17.50 ± 10.88	< 0.001
**Depth of invasion**^a^			0.088
Mucosa	209 (88.6)	1,055 (82.9)	
Sm1	15 (6.4)	112 (8.8)	
Sm2	12 (5.1)	106 (8.3)	
**Atrophy**^b^			0.025
Negative	104 (52.3)	684 (60.7)	
Positive	95 (47.7)	442 (39.3)	
Not applicable^c^	37 (—)	147 (—)	
**Intestinal metaplasia**^b^			< 0.001
Negative	34 (16.0)	409 (34.6)	
Positive	178 (84.0)	772 (65.4)	
Not applicable^c^	23 (—)	85 (—)	
**Differentiation**			0.037
Differentiated	231 (97.9)	1,206 (94.7)	
Undifferentiated	5 (2.1)	67 (5.3)	
**Lauren’s classification**			0.132
Intestinal	231 (97.9)	1,212 (95.5)	
Diffuse	4 (1.7)	26 (2.0)	
Mixed	1 (0.4)	31 (2.4)	
Unknown^c^	0 (—)	4 (—)	

Values are presented as mean ± standard deviation or n (%)

^a^Sm1, tumor invasion <0.5 mm of the muscularis mucosae; Sm2, tumor invasion ≥0.5 mm into the muscularis mucosae

^**b**^The severity of mucosal atrophy and intestinal metaplasia were classified as negative (absent to mild) or positive (moderate to severe)

^c^Excluded from the analysis

### Long-term outcomes

**Fig 3:**
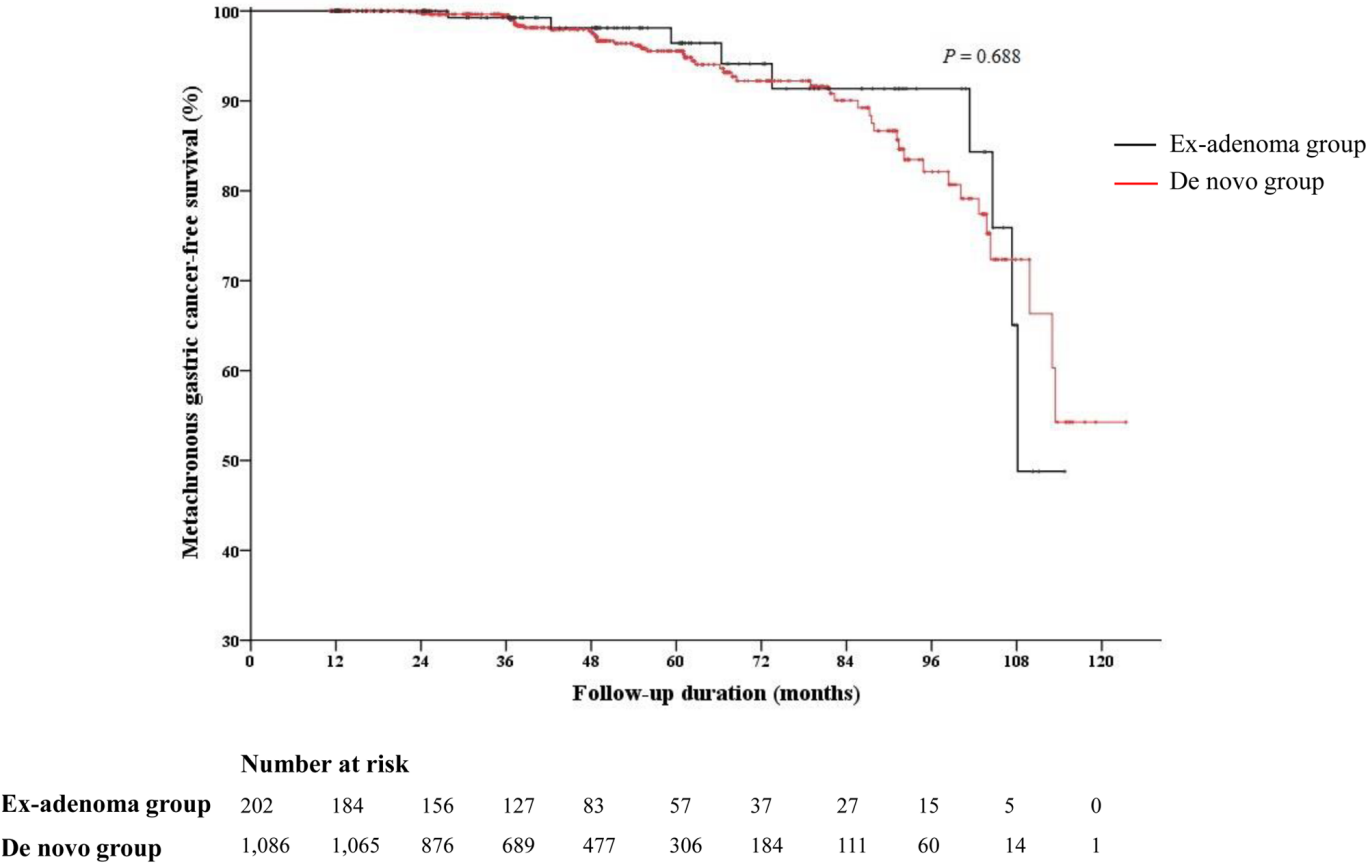
Kaplan-Meier curve of metachronous gastric cancer recurrence. There were no statistically significant differences in the metachronous cancer recurrence rate between the ex-adenoma group and the *de novo* group (*P* = 0.688).

The median follow-up duration was 45.1 months in the ex-adenoma group and 45.4 months in the *de novo* group, which were not statistically different (Table 5). Synchronous gastric neoplasia was significantly more frequent in the ex-adenoma group than in the *de novo* group (21.8% vs. 12.2%, *P* < 0.001), which was mostly due to the significantly higher rate of synchronous gastric adenoma in the ex-adenoma group (14.9% vs. 7.8%, *P* = 0.001), while synchronous gastric carcinoma rates did not differ significantly between the two groups. There was no significant difference in the metachronous cancer recurrence rate during the follow-up period (Fig 3). In the subsequent analysis, there was no statistically significant difference in the characteristics of metachronous cancer (Table 6).

**Table 5:** Follow-up results of the two groups.

	Ex-adenoma group (n = 202)	De novo group (n = 1,086)	*P* value
**Follow-up duration, median (IQR)**	45.1 (24.3–60.9)	45.4 (24.3–61.0)	0.913
**Synchronous gastric neoplasia**^a^	44 (21.8)	133 (12.2)	< 0.001
Synchronous gastric adenoma	30 (14.9)	85 (7.8)	0.001
Synchronous gastric carcinoma	14 (6.9)	48 (4.4)	0.126

Values are presented as median (interquartile range) or n (%)

IQR, interquartile range

^a^Synchronous gastric neoplasia was defined as a gastric adenoma or cancer developed in areas other than the site of primary gastric cancer within 1 year of endoscopic submucosal dissection

**Table 6:** Characteristics of metachronous gastric cancer in two groups.

	Ex-adenoma group (n = 11)	De novo group (n = 65)	*P* value
**Pathologic size, mm**	14.20 ± 5.92	19.31 ± 14.64	0.259
**Differentiation**^a^			0.212
Differentiated	7 (63.6)	54 (83.1)	
Undifferentiated	4 (36.4)	11 (16.9)	
**Depth of invasion**^a^			0.210
Mucosa	8 (72.7)	55 (87.3)	
Beyond mucosa	3 (27.3)	8 (12.7)	

Values are presented as mean ± standard deviation or n (%)

^a^Two patients in the de novo group were excluded from the analysis due to insufficient information

## Discussion

The present study is the first to describe the clinical characteristics of EGC arising from adenoma in comparison with that without pre-existing adenoma. Patients in the ex-adenoma group were older and had a higher rate of *H*. *pylori* infection than those in the *de novo* group. Endoscopic and pathologic size of lesions was greater in the ex-adenoma group than that in the *de novo* group. In addition, atrophy or intestinal metaplasia was more frequent in the adjacent mucosa in the ex-adenoma group. Similar to gastric adenoma, the elevated type lesion was predominant in the ex-adenoma group. The differentiated carcinoma rate was higher in the ex-adenoma group than in the *de novo* group. Considering that EGC arising from adenoma develops from *H*. *pylori*–induced gastritis and progresses through a multistep process over a long period, these characteristics are well-matched with each step of the carcinogenic process.

Premalignant lesions have been identified in various human cancers, including colon, liver, pancreas, breast, uterine cervix, and skin cancers. Among these, the adenoma-carcinoma sequence is a well-established carcinogenic pathway in most colorectal cancers and in specific types of hepatocellular carcinoma [23]. Previous molecular studies showed that alterations in specific genes play crucial roles in the progression of colorectal adenoma to carcinoma [7]. In addition, ex-adenoma colorectal cancers show distinctive clinicopathologic characteristics compared with *de novo* colorectal cancers [24]. Little information is available on the characteristics of gastric cancer developed from adenoma, with only one study showing that EGC with a high microsatellite instability (MSI) mutation rate is associated with co-existing underlying adenoma [25].

In our study, pre-existing adenoma was detected in 15.6% (236/1,509) of endoscopically resected EGCs. These results are difficult to compare with those of previous studies because of the limited amount of data available. A recent study by Jahng *et al*. analyzed the incidence of co-existing underlying adenoma in surgically treated EGCs and showed a rate of 39.7% (29/73) in the MSI-high EGC group vs. 19.9% (29/146) in the non-MSI-high group [25]. A brief retrospective review of medical records of surgically resected EGCs in our institute showed that 6.45% of EGCs were accompanied by pre-existing adenoma. To the best of our knowledge, this is the first study to examine the rate of pre-existing adenoma in EGCs eligible for endoscopic resection. Further study is needed to clarify this issue.

In the present study, *H*. *pylori* infection rates were compared using two methods. *H*. *pylori* infection rates showed marginal differences between the two groups when comparisons were made based on the results of either rapid urease test or histology (Table 1). The establishment of *H*. *pylori* infection induced serial changes in the gastric mucosa resulting from the chronic infection. The spread of the infection led to severe atrophic changes in the gastric mucosa. The development of intestinal metaplasia resulted in a reduction in the bacterial load in the stomach, which could have reduced the yield for *H*. *pylori* infection. Since *H*. *pylori* infection was evaluated only by biopsy and without the use of the urea breath test or serology, which could have increased the risk of false-negative results, a second analysis was performed that included an evaluation of the status of the surrounding mucosa. This analysis showed significant differences between the two groups. The combined results indicated that EGCs that arise from gastric adenomas are more frequently associated with *H*. *pylori*–related chronic inflammation than *de novo* EGCs.

The results of the present study indicated that the rate of metachronous cancer recurrence did not differ significantly between two groups, despite the higher rate of concurrent gastric neoplasms in the ex-adenoma group. *H*. *pylori* infection triggers chronic inflammation of the gastric mucosa, and the normal mucosa adjacent to *H*. *pylori*–infected gastric cancer is also susceptible to the development of a second gastric neoplasm as a result of chronic mucosal damage. This concept of “field cancerization” may explain the high incidence of synchronous or metachronous gastric neoplasms in gastric cancer patients [26]. Our results showing a higher rate of synchronous gastric adenoma in the ex-adenoma group can be explained by the higher possibility of field formation in this group. However, the prevalence of synchronous or metachronous gastric carcinoma did not differ between the two groups, despite a lower number of premalignant lesions expected in the *de novo* group. These results indicate that gastric cancer may occur through different mechanisms in the two groups. Further studies are necessary to elucidate the mechanisms underlying gastric carcinogenesis.

The strength of this study lies in the large number of patients included in the analysis, which makes the results relatively robust. Furthermore, to the best of our knowledge this is the first study to demonstrate the clinicopathologic characteristics and long-term outcomes of EGCs with pre-existing adenoma compared with those of *de novo* EGCs.

The present study had several limitations. First, only EGCs treated with endoscopic resection were included in the analysis, which may have introduced selection bias. Diffuse type gastric cancer accounts for approximately 30–50% of all gastric cancers, whereas the rate was 2% in the present study [27, 28]. In addition, the proportion of intestinal-type carcinomas did not significantly differ between the two groups, although it was higher in the ex-adenoma group. This result is inconsistent with those of previous studies suggesting that intestinal-type carcinoma is associated with adenoma-carcinoma sequence, as indicated by a higher rate of intestinal-type carcinoma in *H*. *pylori*-related gastric carcinogenesis. For the same reason, the depth of invasion and the degree of differentiation in gastric cancer in general may be somewhat different from our results. Careful interpretation is required to avoid selection bias, and a large cohort study targeting all types of gastric cancer is needed to verify our novel findings. Second, this was a retrospective study based on the analysis of medical records and a molecular analysis was not included. In the present study, we were unable to determine whether EGCs in the *de novo* group developed directly from the normal mucosa without an intermediate adenoma step or, if a pre-existing adenomatous component was entirely converted into a carcinoma. The prevalence of *de novo* EGCs may be overestimated.

In conclusion, EGCs arising from adenoma were more closely associated with chronic inflammation caused by *H*. *pylori* infection than EGCs without pre-existing adenoma. Despite differences in the carcinogenic mechanism, both groups showed a high incidence of synchronous and metachronous gastric cancer, underscoring the importance of careful surveillance in all endoscopically resected EGCs.
